# Association between periodontal disease and atherosclerosis: a bibliometric analysis

**DOI:** 10.3389/fcvm.2024.1448125

**Published:** 2024-11-14

**Authors:** Haoxiang Chang, Yahui Wang, Ziqi Zhang, Zhongqian Mi, Xinjie Qiu, Miaomiao Zhao, Chong Wang, Xue Bai, Xiuyun Ren

**Affiliations:** ^1^ Shanxi Medical University School and Hospital of Stomatology, Taiyuan, China; ^2^ Shanxi Province Key Laboratory of Oral Diseases Prevention and New Materials, Taiyuan, China

**Keywords:** periodontal disease (PD), atherosclerosis, bibliometrics, inflammation, Porphyromonas gingivalis (P. gingivalis), mechanisms

## Abstract

**Background:**

In recent years, the relationship between periodontal disease (PD) and atherosclerosis (AS) has garnered significant scholarly attention. Chronic inflammation induced by PD may promote the onset and progression of AS through multiple mechanisms. Given the increasing global incidence of both PD and AS, which adversely affects patients’ quality of life and longevity, further investigation into the interplay between PD and AS is of substantial clinical importance. This study aims to conduct a comprehensive analysis of the association between PD and AS using bibliometric methods.

**Methods:**

Articles and reviews on the association between PD and AS were retrieved from the Web of Science Core Collection (WOSCC) on June 1, 2023. Bibliometric and knowledge mapping analyses were conducted using CiteSpace [6.3.R1 (64-bit) Advanced].

**Results:**

Through a bibliometric analysis of the literature published between 2000 and 2023 on the PD-AS relationship, we identified 1,572 relevant studies. The results show a steadily increasing number of studies on this topic annually, with a significant upward trend after 2002. Keyword analysis reveals “atherosclerosis,” “periodontal disease,” “cardiovascular disease,” “Porphyromonas gingivalis,” and “periodontal pathogens” as research hotspots. Collaboration network analysis highlights the United States as the primary contributor to research in this field. Co-citation analysis shows that *J Periodontol*, *J Clin Periodontol*, and *Circulation* are the most frequently cited journals, reflecting their support for research in this area. Document co-citation analysis identifies several high-impact studies focusing on how systemic inflammation induced by periodontitis promotes AS. This study uncovers development trends and research hotspots in the PD-AS relationship and emphasizes the role of international collaboration and high-impact journals in advancing this field. These insights provide valuable references and guidance for future research.

**Conclusion:**

PD and AS are global epidemics causing significant distress and imposing a substantial burden. Research on the PD-AS relationship is evolving rapidly and continues to be a critical focus. Elucidating the mechanisms linking PD and AS represents an emerging trend, providing valuable references for future studies.

## 1 Introduction

Periodontal disease (PD) is a group of inflammatory conditions affecting the supporting structures of the teeth (gingiva, alveolar bone, periodontal ligament), potentially leading to tooth mobility and loss (1). It has been reported that approximately 50% of the global population suffers from PD, significantly impacting their nutritional intake, quality of life, and self-esteem (2). The onset of PD is primarily caused by dysbiosis of the oral microbiome, which may lead to immune system dysfunction and a chronic inflammatory phenotype (3).

Atherosclerosis (AS) is a chronic disease characterized by the accumulation of lipid plaques, primarily composed of cholesterol and fats, on the inner walls of arteries. These plaques cause arterial hardening and narrowing, reducing blood flow and potentially leading to vascular occlusion. The pathogenesis of atherosclerosis involves the subendothelial accumulation of lipids, an inflammatory response, smooth muscle cell proliferation, and plaque fibrosis (4). In recent decades, the relationship between periodontal disease (PD) and AS has attracted increasing attention from researchers. Chronic inflammation induced by PD may promote the onset and progression of atherosclerosis through various mechanisms: (1) Release of Inflammatory Mediators: Periodontal inflammation leads to the release of cytokines and other inflammatory mediators into the bloodstream, elevating systemic inflammation. These inflammatory factors play a crucial role in the development of atherosclerosis (5). (2) Bacterial Dissemination: Periodontal pathogens, such as Porphyromonas gingivalis (P.g), can disseminate through the bloodstream and directly infect atherosclerotic plaques, exacerbating their formation (6). (3) Immune Response: Chronic periodontal infection may trigger a host immune response, causing the immune system to attack lipid deposits within the arterial walls, thereby accelerating plaque formation and progression (7). Given the rising global prevalence of both PD and AS, and their impact on patients’ quality of life and longevity, further research into the interplay between PD and AS holds significant clinical importance.

Bibliometrics is a discipline that applies mathematical and statistical methods to analyze books, articles, and other publications. Its purpose is to assess and track the quantity and quality of scientific research activities, as well as to understand the structure, development, and trends in academic research (8). Over the past decade, bibliometrics has been widely applied in the field of medical research, including studies on periodontal disease (PD), atherosclerosis (AS), cancer, COVID-19, and metabolic diseases. Recently, Song et al. conducted a bibliometric analysis on the relationship between PD and coronary heart disease (CHD). However, their study did not specifically focus on atherosclerosis but rather encompassed the entire field of coronary artery diseases (9).

This study aims to conduct a comprehensive bibliometric analysis of the relationship between periodontal disease (PD) and atherosclerosis (AS). We will summarize the knowledge base, research hotspots, and development trends in this field, thereby laying a solid foundation for future research. Further elucidation of the PD-AS relationship may significantly impact the diagnosis and treatment of both diseases, promoting advancements in clinical practice. This research will not only assist researchers and experts in gaining a deeper understanding of the association between periodontal disease and atherosclerosis but also ultimately benefit patients with these conditions by improving their treatment outcomes and quality of life.

## 2 Methods

### 2.1 Data source and search strategy

This study utilized the Web of Science Core Collection (WOSCC) database (https://www.webofscience.com) as the data source, widely regarded as the premier database for bibliometric analysis (10). The search query employed was “TS = (“periodontal disease” OR periodont* OR gingiv* OR “tooth loss” OR “tooth migration” OR “tooth mobility”) AND TS = (atherosclerosis OR atheroscleroses OR atherogenesis)”. All retrieved documents were independently evaluated by two reviewers (HXC and YHW) based on titles, abstracts, and keywords to exclude studies unrelated to PD and AS. In cases of disagreement between the two reviewers, the judgment of a third reviewer (ZZQ) was deemed final. To minimize bias from database updates, the literature search was completed in a single day (2024-06-01).

### 2.2 Inclusion criteria

Publications meeting the following criteria were included in the subsequent analysis, while all other articles were excluded:

(1) Articles and review papers related to the fields of PD and AS;
(2) Articles and review papers written in English;
(3) Articles and review papers published between 2000 and 2023.

### 2.3 Exclusion criteria

Non-English publications, publication types other than articles and review papers, and publications dated before 2000 or after 2023 were excluded from the bibliometric analysis.

### 2.4 Data collection and data analysis

The retrieved papers were exported in the form of “Full Record and Cited References” and saved in “Plain Text”. In addition, these files were named “download_.txt”.

Microsoft Office Excel 2019 was used for data management and analysis of annual publications. Additionally, we utilized CiteSpace [6.3.R1 (64-bit) Advanced] to analyze these data and visualize scientific knowledge maps.

CiteSpace, developed by Chaomei Chen, is a Java-based citation visualization software that provides an experimental platform for exploring new ideas and comparing existing methods. It is one of the most widely used visualization analysis tools in bibliometrics. CiteSpace can analyze the underlying literature from multiple perspectives, identify research hotspots and trends in specific fields, and present them visually. Knowledge maps generated by CiteSpace can help researchers intuitively understand research hotspots and their evolution, and predict research and development trends in areas of interest (11).

## 3 Result

### 3.1 General information

**Figure 1:**
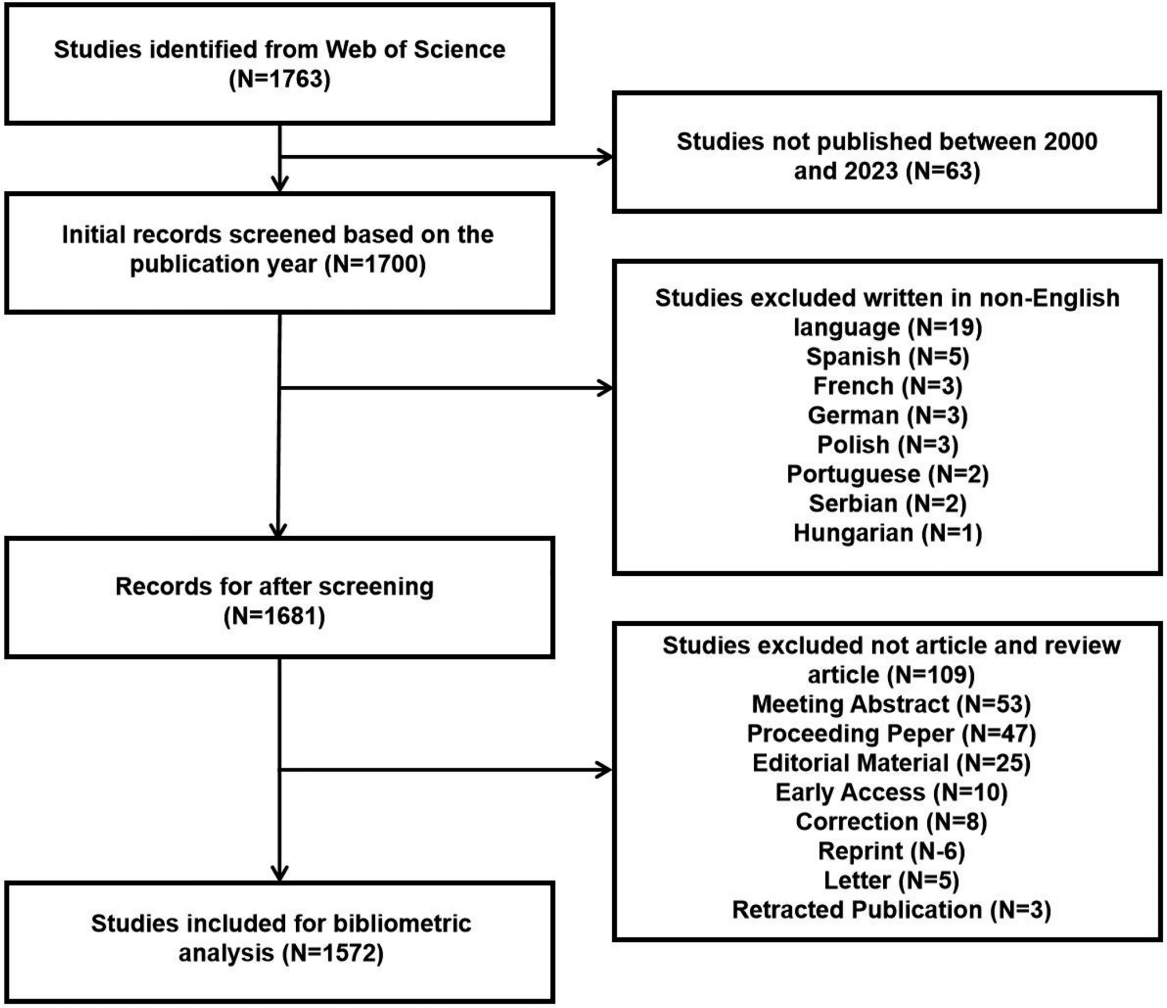
Flowchart of the search strategy.

**Figure 2:**
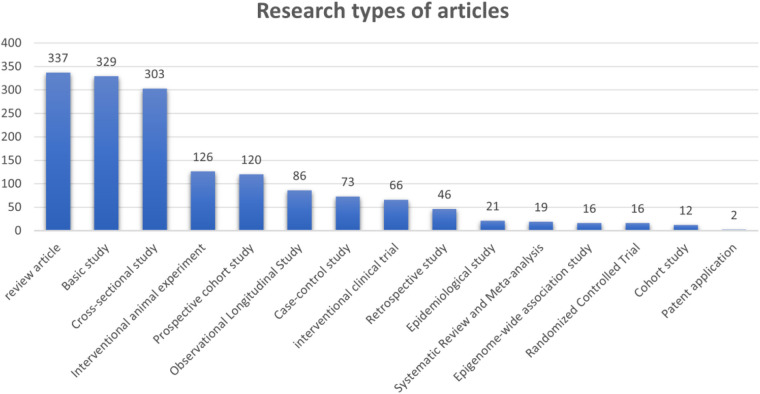
Research types of articles.

**Figure 3:**
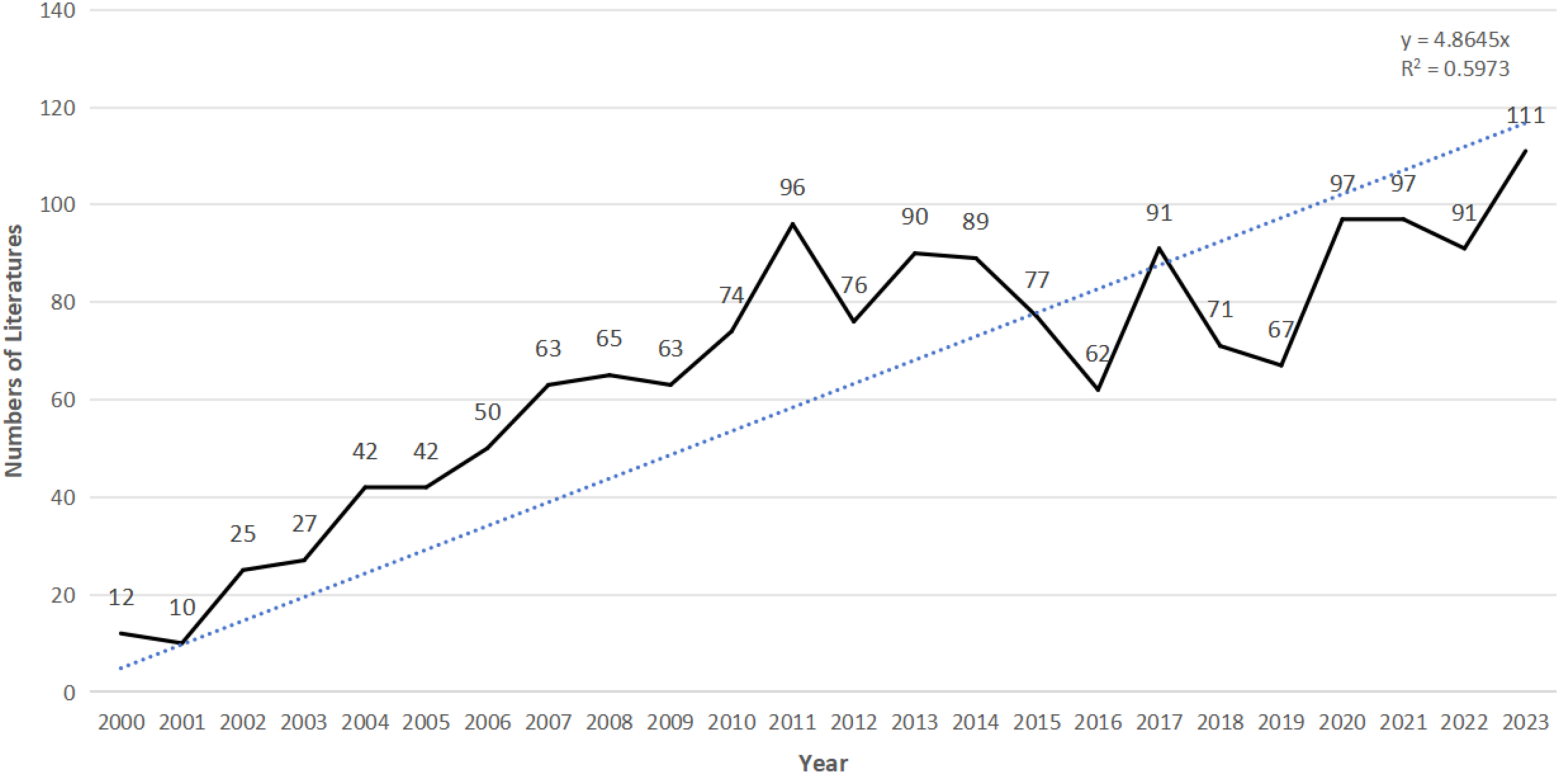
The number of published studies over time.

The literature search retrieved a total of 1,763 publications. After screening, 1,572 publications were included in the subsequent bibliometric analysis, comprising 1,235 research articles (1,235/1,572, 78.562%) and 337 review articles (337/1,572, 21.438%) (Figure 1). The research types of these 1,572 articles are shown in Figure 2. From 2000 to 2001, the annual number of publications in this field remained below 20 per year. However, since 2002, the annual publication count has shown a significant upward trend (*R*^2^ = 0.5973), peaking at 111 publications in 2023 (Figure 3). Figure 3 illustrates that from 2002 to 2011, the publication volume increased rapidly, while from 2012 to 2023, it exhibited a slower growth trend, reaching its peak in 2023. This trend indicates that since 2002, there has been a growing interest among scholars in the relationship between PD and AS. The sustained high level of research activity from 2012 to 2023 reflects the continued international focus on this field. Overall, research on PD and AS has experienced several significant growth phases over the past 23 years, particularly the rapid increase post-2002, highlighting the academic community's increasing emphasis on this area. The publication volume in 2023, reaching 111 papers, demonstrates the current high level of research activity.

The consistent year-by-year growth trend suggests that research on the association between PD and AS is gaining increasing attention, indicating that more research outcomes are likely to emerge in the future. This will provide stronger scientific evidence for the prevention and treatment of related diseases.

### 3.2 Cooperation network analysis

#### 3.2.1 Distribution of countries/regions

**Figure 4:**
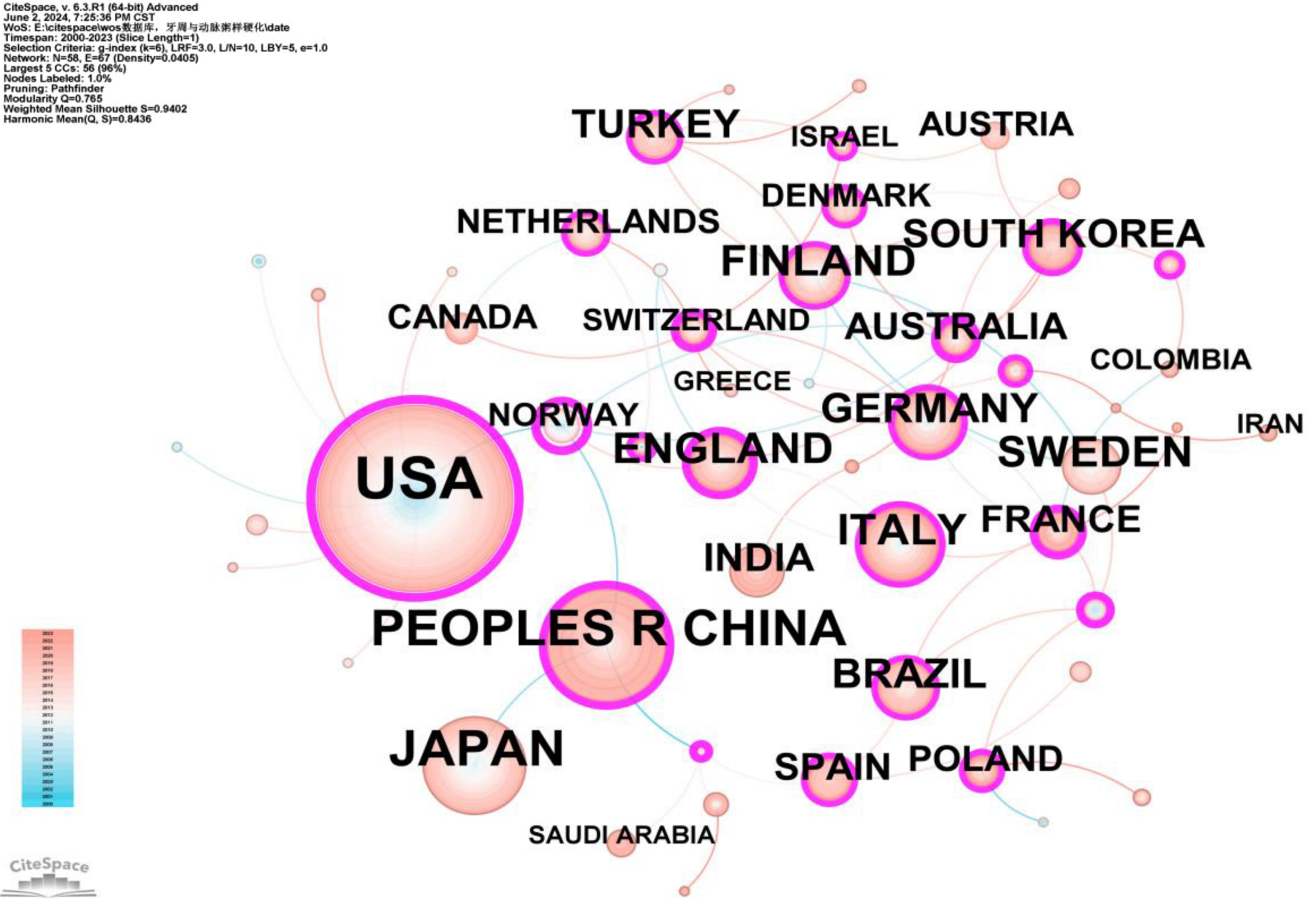
Cooperation network map of countries/regions. After normalizing the sample literature (1,572 publications), the data was imported into CiteSpace for analysis. The selected time period spanned from 2000 to 2023, and the analysis focused on the “country” level to construct a co-occurrence network map of countries researching the relationship between PD and AS. The size of the nodes represents the number of publications, while the links between nodes indicate collaborative efforts. Different colors represent the years of cooperation. The outermost purple ring denotes the level of centrality, with high centrality nodes considered key points in the research field. The network parameters are as follows: g-index (k = 5), *N* = 58 (number of network nodes), E = 67 (number of connections), and density = 0.0405 (network density).

The 1,572 publications originated from 58 countries, with the United States contributing over one-quarter of the total publications (476/1,572, 29.14%), followed by Japan and China (Supplementary Table S1). This highlights the significant influence of the United States in this field. Additionally, Norway achieved the highest betweenness centrality (BC) value of 0.68, indicating a high level of collaboration with other countries (Supplementary Table S2). The visualization of country/region collaborations shows that Norway's close collaborators primarily include major countries such as the United States, China, the United Kingdom, and Australia (Figure 4).

#### 3.2.2 Distribution of institutions

**Figure 5:**
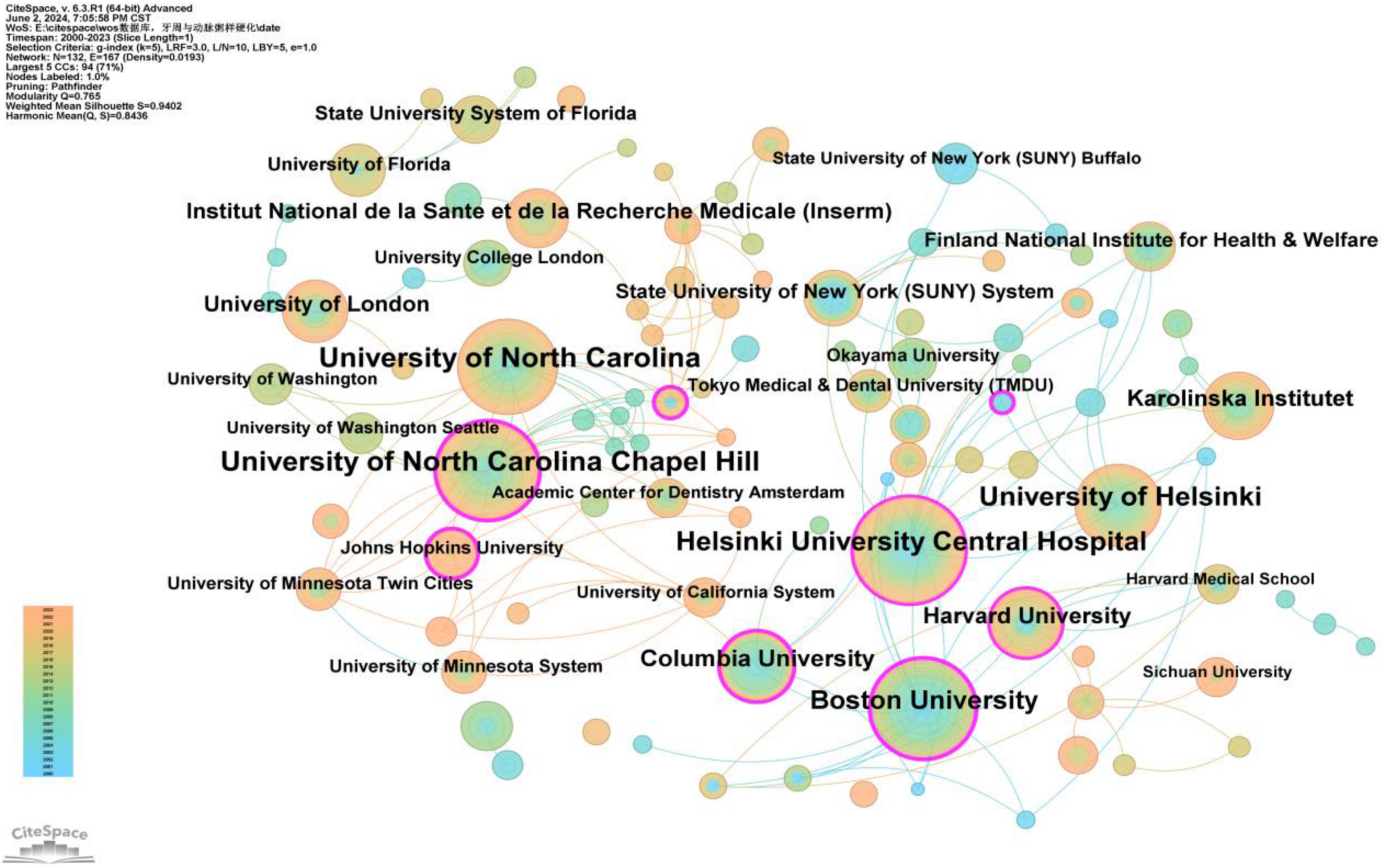
Cooperation network map of institutions. After normalizing the sample literature (1,572 publications), the data was imported into CiteSpace for analysis. The selected time period spanned from 2000 to 2023, with the analysis focused on the “institution” level to construct a co-occurrence network map of institutions researching the relationship between PD and AS. Node size represents the number of publications, while the links between nodes indicate collaborative efforts. Different colors represent the years of cooperation. The outermost purple ring denotes the level of centrality, with high centrality nodes considered key points in the research field. The network parameters are as follows: g-index (k = 5), *N* = 132 (number of network nodes), E = 167 (number of connections), and Density = 0.0193 (network density).

Authors from 132 institutions have published research in this field. The institutions with the highest number of publications were the University of North Carolina and the University of North Carolina at Chapel Hill, each with a frequency of 74 (Supplementary Table S3). Two institutions had a betweenness centrality (BC) exceeding 0.4: Boston University (0.47) and Columbia University (0.4) (Supplementary Table S4). Figure 5 reveals the following characteristics of international research institutions and their collaborations in the study of the relationship between PD and AS:

1. Key Institutions: In terms of publication volume, several institutions have emerged as key players in researching the PD-AS relationship. The largest nodes, indicating the highest publication volumes, are the University of North Carolina and the University of North Carolina at Chapel Hill, followed by the University of Helsinki, Helsinki University Central Hospital, Boston University, Columbia University, Harvard University, Karolinska Institutet, University of London, Institut National de la Santé et de la Recherche Médicale (Inserm), State University of New York (SUNY) System, and the Finland National Institute for Health & Welfare. These institutions constitute the core forces in the research on the relationship between PD and AS.
2. Research Trends and Geographical Distribution: Most of the top 20 institutions in terms of publication numbers are based in the USA, underscoring the significant influence of American institutions in this field. This is consistent with the results shown in Figure 3. The geographical distribution of research institutions is relatively broad, with high research interest across various international regions. Notably, significant research activity is seen in New York, Massachusetts, and Maryland in the USA, as well as in China, Sweden, and the United Kingdom. This global distribution highlights the widespread importance and attention to research on the relationship between PD and AS.

#### 3.2.3 Authors and co-cited authors

**Figure 6:**
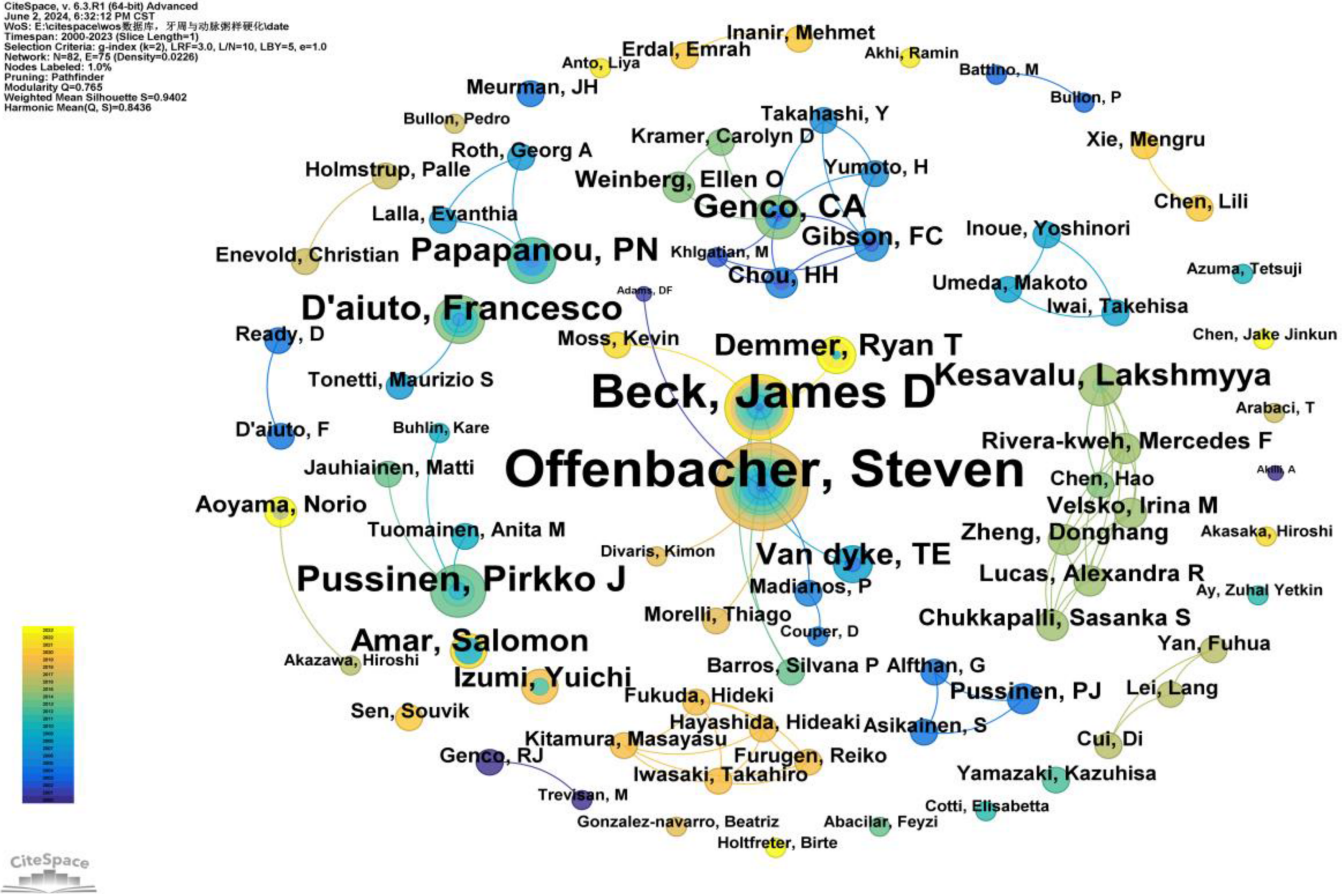
Cooperation network map of authors. After normalizing the sample literature (1,572 publications), the data was imported into CiteSpace for analysis. The selected time period spanned from 2000 to 2023, with the analysis focused on the “author” level to construct a co-occurrence network map of authors researching the relationship between PD and AS. Node size represents the number of publications by each author, while the links between nodes indicate collaborative efforts. Different colors represent the years of cooperation. The network parameters are as follows: g-index (k = 2), *N* = 82 (number of network nodes), E = 75 (number of connections), and Density = 0.0226 (network density).

A total of 82 authors have contributed to publications on the relationship between PD and AS. Only four authors have published more than 10 articles: Offenbacher, Steven (40 articles), Beck, James D. (28 articles), Pussinen, Pirkko J. (12 articles), and D'Aiuto, Francesco (11 articles) (Supplementary Table S5). As shown in Figure 6, the number of connections is fewer than the number of nodes, indicating a relatively low density and suggesting limited collaboration among key researchers in this field. Most researchers work independently and rarely co-author papers. Notably, there is closer collaboration among certain influential authors, primarily Offenbacher, Steven, and Beck, James D., who have formed a substantial research network within this field. Beck, James D., in particular, has maintained a consistent presence in this research area from his first publication in 2003 (12) to 2023 (13–18). Moreover, new authors publish their first papers in this field each year, demonstrating the sustained interest and vitality of research in this area (Figure 6).

**Figure 7:**
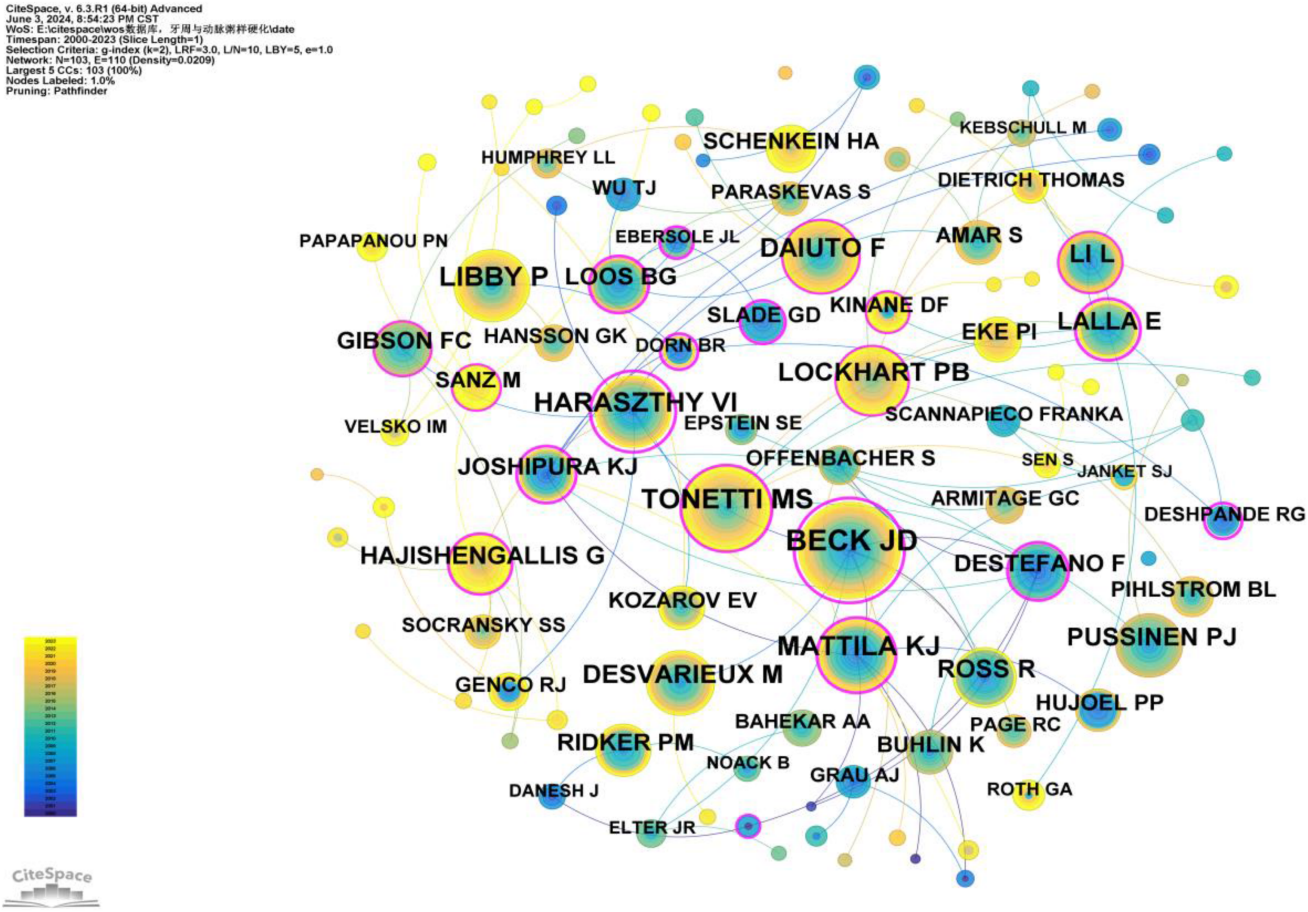
Co-citation network map of authors. After normalizing the sample literature (1,572 publications), the data was imported into CiteSpace for analysis. The selected time period spanned from 2000 to 2023, and the analysis focused on the “Author Co-citation Network” to construct a co-citation network map of authors researching the relationship between PD and AS. Node size represents the number of citations each author received, while the links between nodes indicate co-citations within the same paper. Different colors represent the years in which the citations occurred. The network parameters are as follows: g-index (k = 2), *N* = 103 (number of network nodes), E = 110 (number of connections), and density = 0.0209 (network density).

Co-citation refers to two (or more) authors being cited together in one or more papers. As shown in Supplementary Table S6, the top 10 co-cited authors have been cited over 190 times. The most frequently co-cited author is BECK JD (*n* = 548), followed by TONETTI MS (*n* = 360), MATTILA KJ (*n* = 291), HARASZTHY VI (*n* = 288), and LIBBY P (*n* = 285). Additionally, 11 authors have a centrality greater than 0.40 (see Supplementary Table S7), with HARASZTHY VI (1.27) having the highest centrality, followed by BECK JD (1.12), both exceeding 1.00. These highly central co-cited authors are depicted with purple rings in Figure 7, indicating their significant bridging roles within the research network.

### 3.3 Keyword analysis

Keywords provide a concise summary of an article, and analyzing them can identify research hotspots and emerging trends. A total of 150 keywords were identified, with the top five high-frequency keywords being: atherosclerosis (684 occurrences, BC = 0.33), periodontal disease (462 occurrences, BC = 0.23), cardiovascular disease (412 occurrences, BC = 0.08), Porphyromonas gingivalis (370 occurrences, BC = 0.33), and coronary heart disease (328 occurrences, BC = 0.6). The high frequency of these keywords indicates that they are major focal points of research (Supplementary Table S8).

Additionally, the top five keywords ranked by betweenness centrality (BC) are: infection (BC = 0.66), coronary heart disease (BC = 0.6), dental disease (BC = 0.43), periodontal pathogens (BC = 0.42), and atherosclerosis (BC = 0.33). The high centrality of these keywords suggests that they play crucial bridging roles in the research network, connecting a wide range of other research topics (Supplementary Table S9).

**Figure 8:**
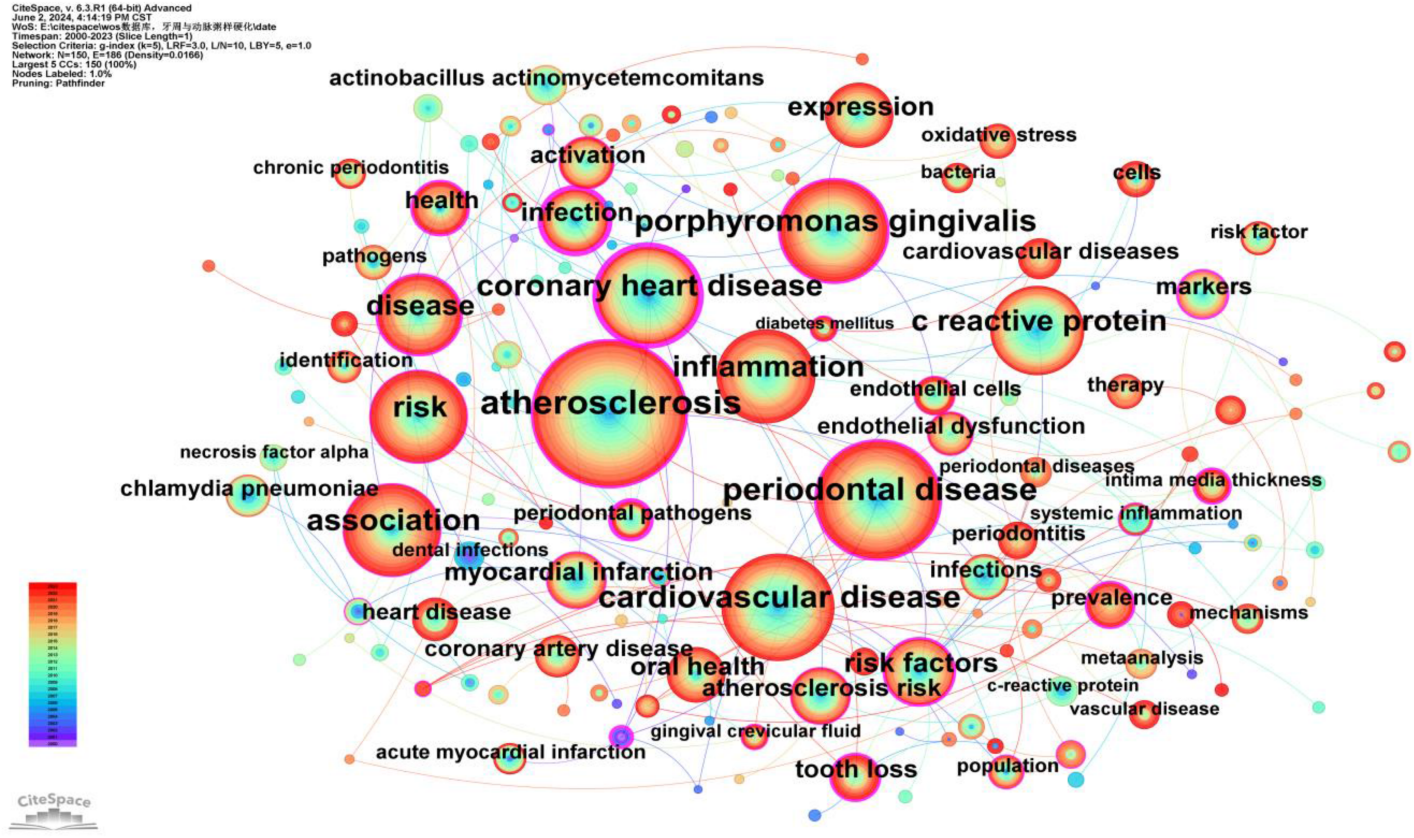
Keywords co-occurrence network map. After normalizing the sample literature (1,572 publications), the data was imported into CiteSpace for analysis. The selected time period spanned from 2000 to 2023, focusing on the “keywords” level to construct a co-occurrence network map of keywords related to the relationship between PD and AS. Node size represents the frequency of keyword occurrences, while links between nodes indicate co-occurrence between different keywords. Different colors represent the years of occurrence. The outermost purple ring denotes the level of centrality, with high centrality nodes considered key points in the research field. The network parameters are as follows: g-index (k = 5), *N* = 150 (number of network nodes), E = 186 (number of connections), and density = 0.0166 (network density).

**Figure 9:**
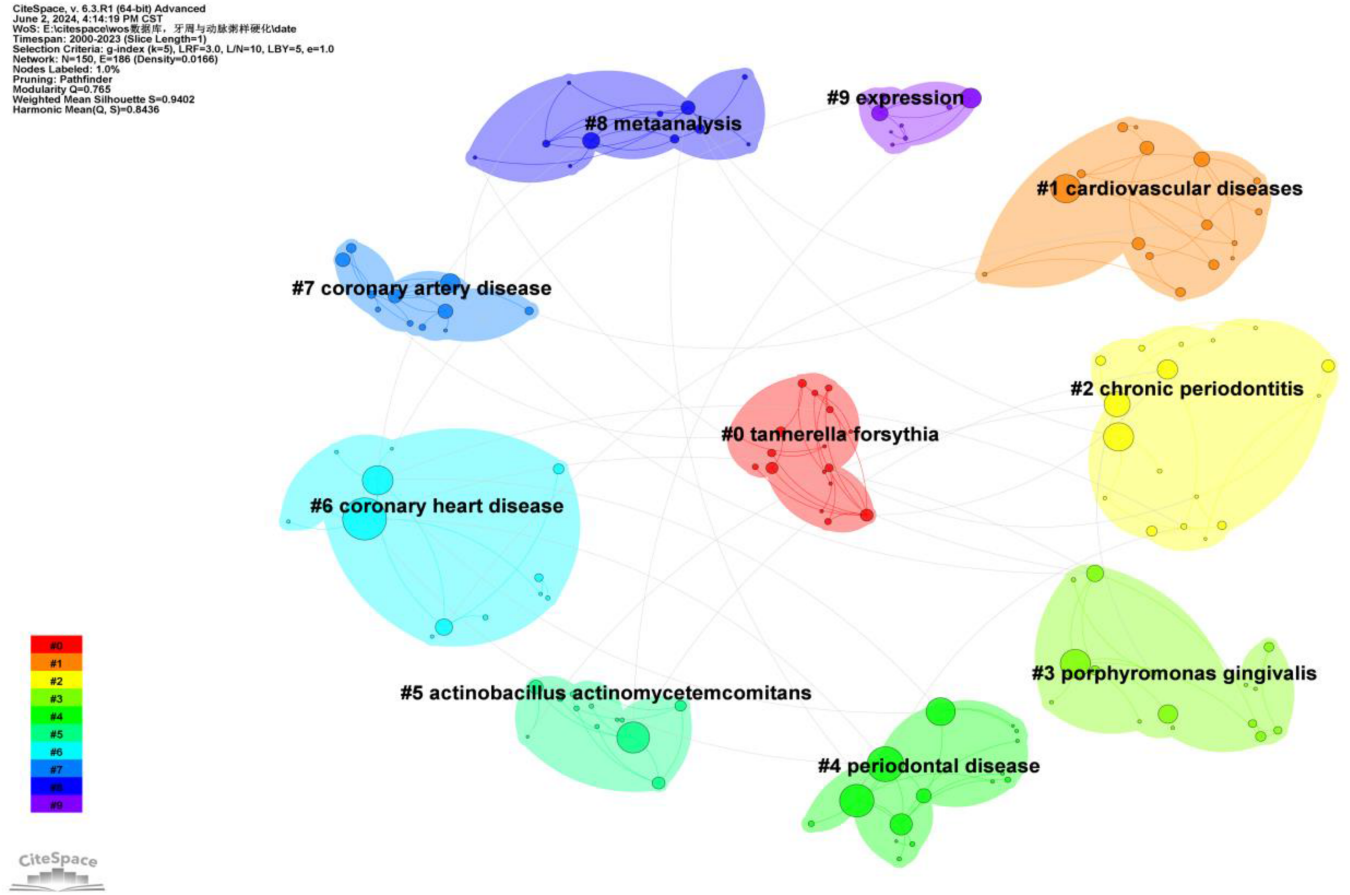
Cluster analysis of keywords.

To better reflect the research hotspots and trends in this field, we used CiteSpace software to generate a high-frequency keyword co-occurrence network (Figure 8) and a cluster network map (Figure 9).

**Figure 10:**
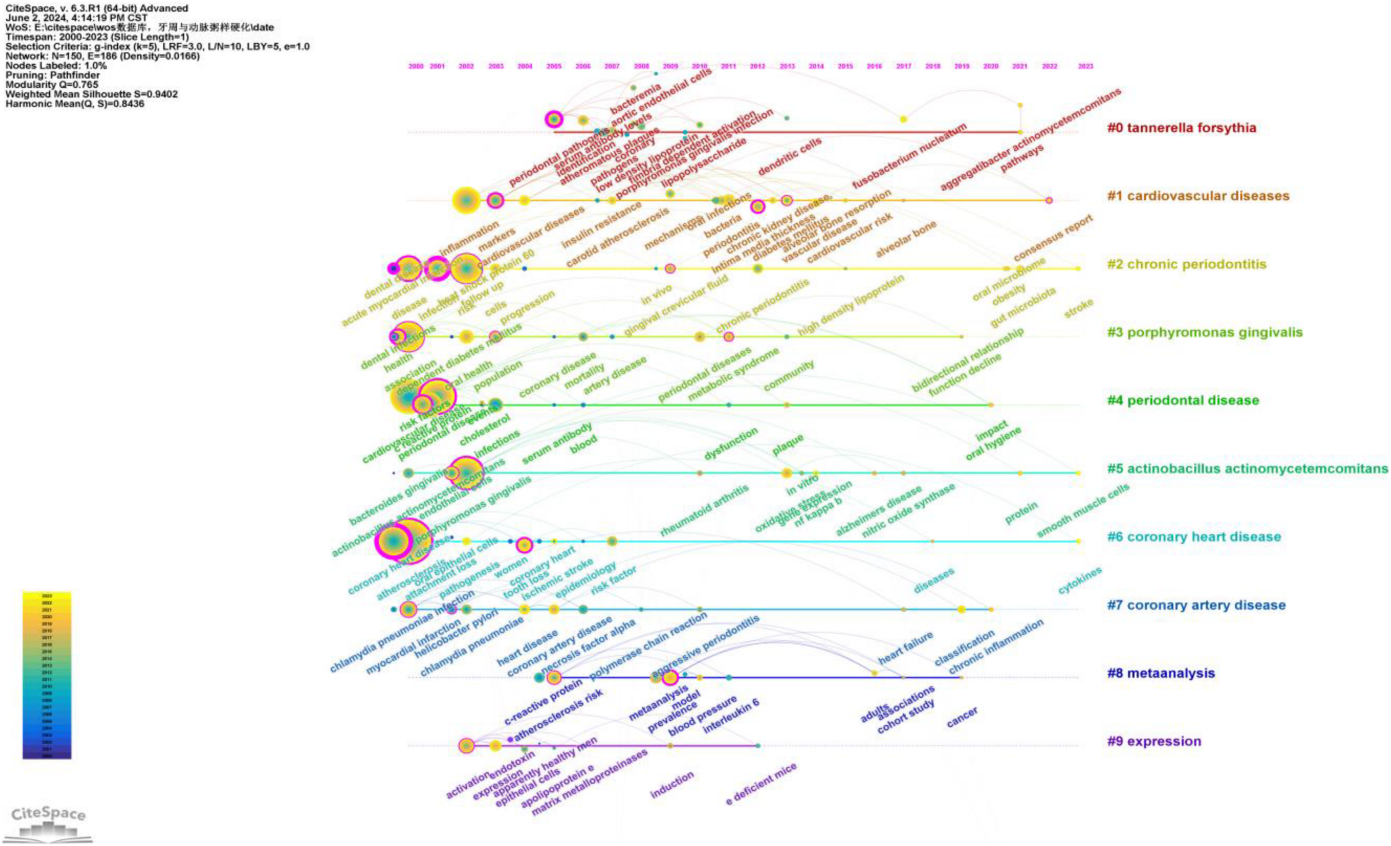
Keywords timeline network map. This figure illustrates the evolutionary trends of keywords related to periodontal disease and atherosclerosis from 2000 to 2023. In the figure, nodes represent keywords, with the size of each node indicating the frequency of the keyword, and the color representing the time of the keyword's first appearance. Links between nodes indicate co-occurrence relationships between the keywords.

As shown in Figure 9, a total of ten clusters were identified: #0 Tannerella forsythia, #1 cardiovascular diseases, #2 chronic periodontitis, #3 Porphyromonas gingivalis, #4 periodontal disease, #5 Actinobacillus actinomycetemcomitans, #6 coronary heart disease, #7 coronary artery disease, #8 meta-analysis, and #9 expression. The occurrence time, frequency, and co-occurrence relationships of keywords within each cluster are illustrated in Figure 10. Supplementary Table S10 lists the main keywords in each cluster.

Cluster #0 includes keywords such as CD204, receptor-deficient mice, periodontal bacteria, pregnancy complications, and scaling and root plaining Analysis of these keywords indicates that this cluster primarily focuses on the study of periodontal bacteria and their effects on systemic health, particularly pregnancy complications and periodontal treatment methods such as scaling and root plaining.

Cluster #1 includes keywords such as leukocyte activation, host-pathogen interactions, leukocytes, CD62P, and intimal hyperplasia. These keywords suggest that this cluster focuses on the role of leukocyte activation and host-pathogen interactions, particularly in relation to intimal hyperplasia. This indicates that periodontal infections may influence the development of atherosclerosis through immune responses.

Cluster #2 includes keywords such as abdominal aortic aneurysm, HBD-2, host response, HBD-3, and adhesion molecules. These keywords indicate that this cluster involves research on abdominal aortic aneurysm and host responses, particularly the role of adhesion molecules. These studies suggest that periodontal pathogens influence the development of atherosclerosis through adhesion molecules.

Cluster #3 includes keywords such as end-stage kidney disease, colonization, machine learning, PAD, and blood cells. These keywords indicate that this cluster focuses on research related to end-stage kidney disease and vascular diseases such as peripheral arterial disease (PAD), as well as the application of machine learning in such studies. This suggests that periodontal disease may affect kidney function and vascular health through systemic inflammation.

Cluster #4 includes keywords such as carotid endarterectomy, duplex sonography, dentition, estimated glomerular filtration rate (eGFR), and cause-specific mortality. These keywords suggest that this cluster primarily involves studies on the use of carotid endarterectomy and sonography in assessing atherosclerosis, as well as the relationship between dental health and cause-specific mortality.

Cluster #5 includes keywords such as NOX2, NOX4, apolipoproteins, lymphatic circulation, and HSP10. Analysis of these keywords indicates that this cluster focuses on the role of oxidoreductases (such as NOX2 and NOX4) and apolipoproteins in atherosclerosis. These studies suggest that periodontal disease influences atherosclerosis through oxidative stress and lipid metabolism pathways.

Cluster #6 includes keywords such as acid sphingomyelinase, density, attachment loss, carotid calcification, and etiology. These keywords suggest that this cluster involves research on the role of acid sphingomyelinase in atherosclerosis, particularly the relationship between carotid calcification and periodontal attachment loss.

Cluster #7 includes keywords such as atherosclerosis/etiology, serum fibrinogen, TEM, coronary artery stenosis, and cardiolipin. These keywords indicate that this cluster focuses on the etiology of atherosclerosis, including serum fibrinogen and coronary artery stenosis. These studies suggest that periodontal disease may influence the development of atherosclerosis through inflammatory markers in the blood.

Cluster #8 includes keywords such as epigenome-wide association study (EWAS), behavior, periomedicine, and Parkinson's disease. These keywords suggest that this cluster focuses on epigenome-wide association studies and the application of behavioral medicine in periodontal disease and atherosclerosis research, particularly the correlation with neurological disorders such as Parkinson's disease.

Cluster #9 includes keywords such as murine model, cytokine induction, oral submucous fibrosis, glucose, and lncRNA. Analysis of these keywords indicates that this cluster involves studies on cytokine induction in murine models, oral submucous fibrosis, glucose, and long non-coding RNAs (lncRNAs), suggesting that periodontal disease may influence atherosclerosis through multiple biological pathways.

As shown in Figure 10, research on the relationship between periodontal disease and atherosclerosis has deepened and expanded over the past two decades. Research hotspots have evolved from early studies on pathogens and basic mechanisms to more complex areas such as the microbiome, systemic inflammation, and gene expression. These studies provide valuable insights into how periodontal disease impacts atherosclerosis and point to future research directions.

**Figure 11:**
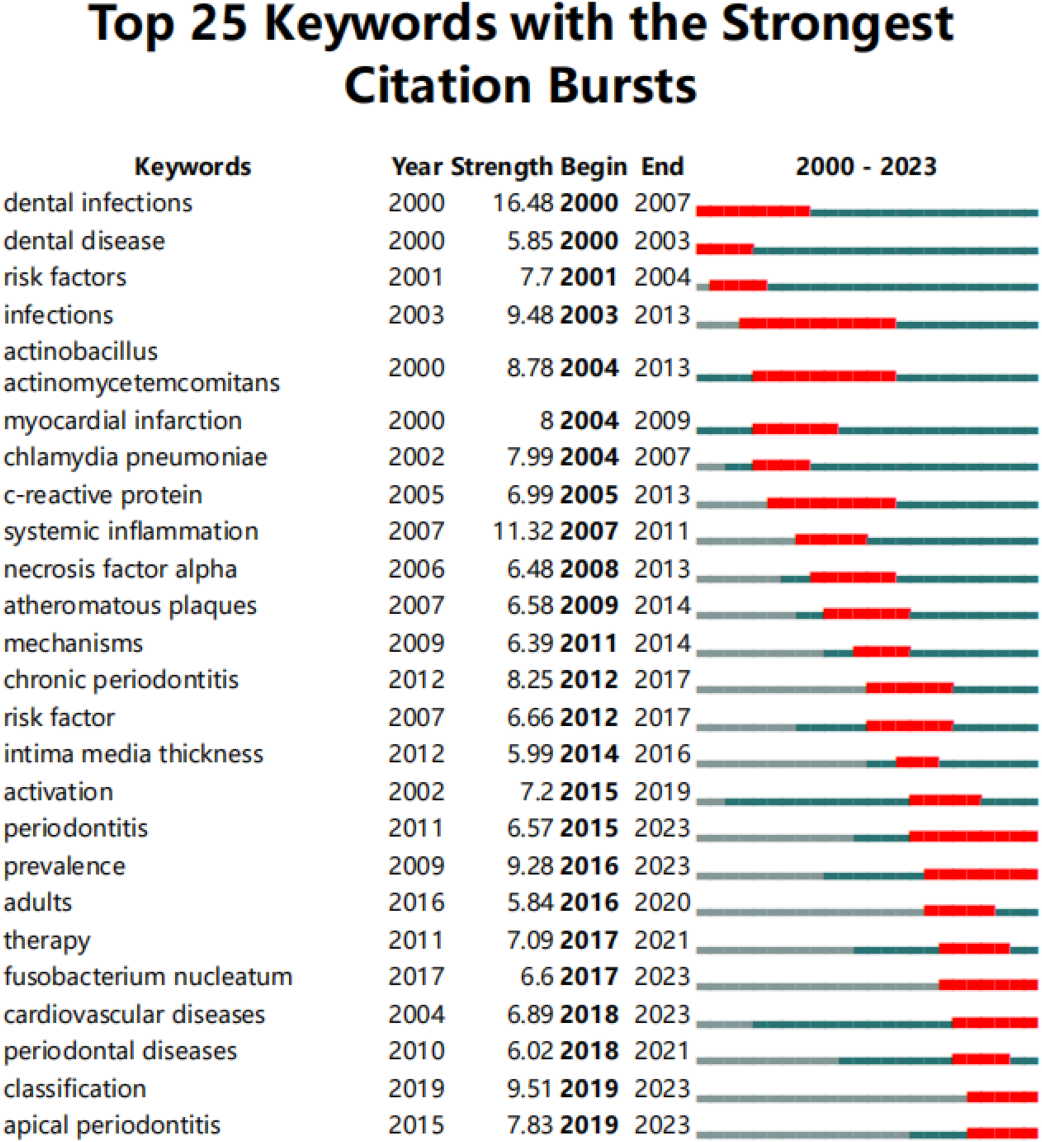
Top 25 keywords with the strongest citation bursts. The red line represents years when keywords burst, and the blue line indicates years when keywords were used less frequently. Burst strength reflects the occurrence times of keywords in a certain period. Each keyword burst lasts for a minimum duration of 3 years.

Burst keyword analysis identifies emerging research fronts and trends by examining the temporal distribution of keywords and detecting those with high change rates and rapid growth. As shown in Figure 11, the keyword “infection” had the longest burst duration, while “dental infections” exhibited the highest burst strength. Additionally, “classification” and “apical periodontitis” are the most recent burst keywords, emerging since 2019.

Time zone maps are composed of a series of vertical bands representing different time periods. These maps primarily display the evolution of knowledge from a temporal perspective, clearly illustrating the relationships and updates of documents. The time zones are arranged sequentially from left to right, highlighting the research frontiers in relation to their knowledge base. The “Time Zone” function in CiteSpace not only displays cited keyword information but also shows citation information in the background data, facilitating the connection between research frontiers and their foundational knowledge.

**Figure 12:**
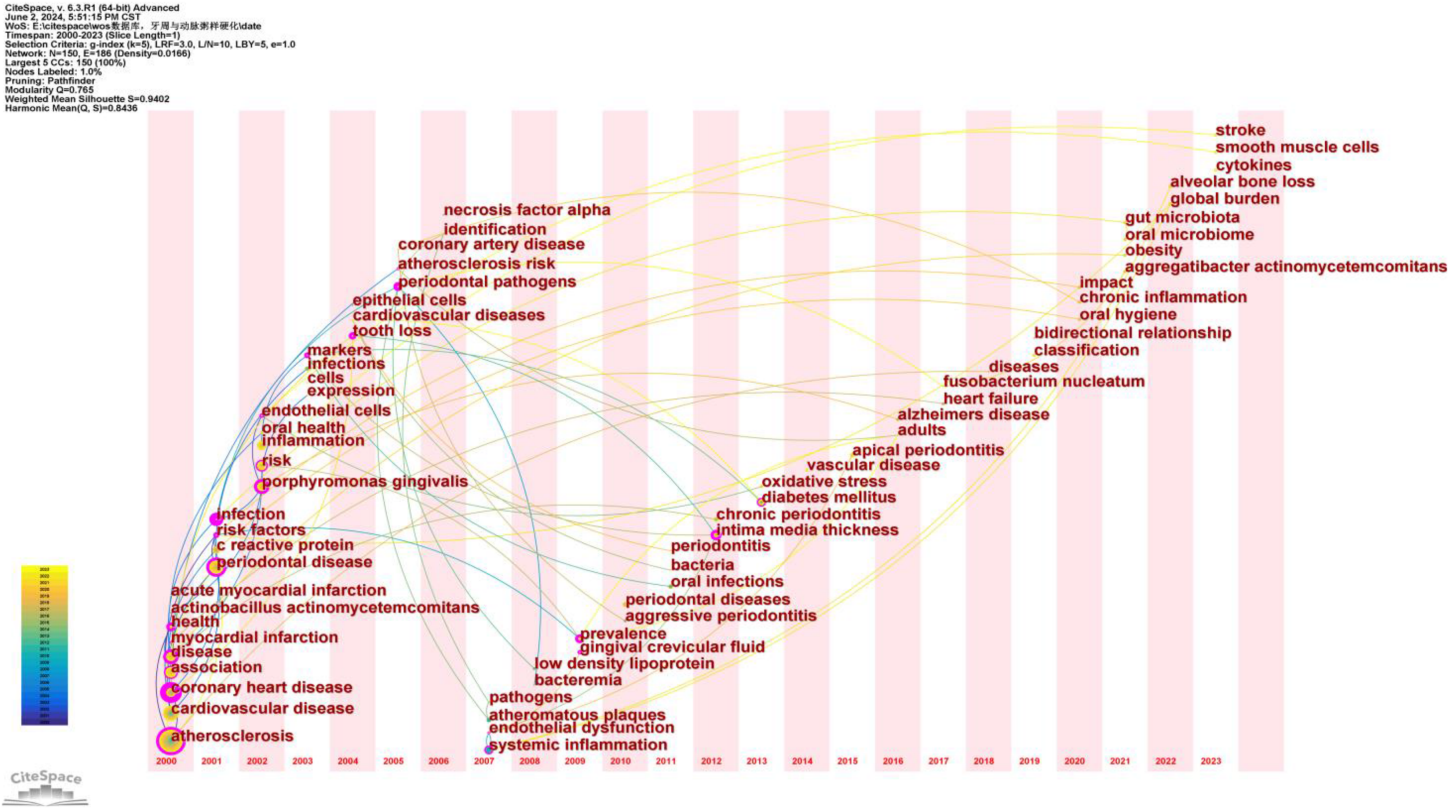
Keyword time zone map. In this figure, nodes represent keywords, with the size of each node indicating the frequency of the keyword, and the color representing the time of the keyword's first appearance. Links between nodes indicate co-occurrence relationships between the keywords.

After normalizing the sample literature (1,572 publications), the data was imported into CiteSpace for analysis. The selected time period was from 2000 to 2023, with the analysis focused on “keywords,” resulting in a keyword co-occurrence map. By using the Time Zone function, we generated a time zone view of the association between PD and AS from 2000 to 2023, as shown in Figure 12. This figure clearly illustrates the evolution of research hotspots and frontiers in this field, providing valuable insights for analyzing the dynamics of the association between PD and AS.

Based on the timeline in Figure 12, the research can be broadly divided into three stages:

Early Research (2000–2005): Primary keywords include “atherosclerosis,” “cardiovascular disease,” “coronary heart disease,” “periodontal disease,” and “infection.” During this period, research focused on the fundamental relationship between atherosclerosis and cardiovascular diseases, with particular attention to periodontal disease as a potential contributing factor. Keywords such as “atherosclerosis” and “periodontal disease” indicate early interest in the association between these two conditions.

Mid-term Research (2006–2015): Primary keywords include “endothelial dysfunction,” “systemic inflammation,” “low-density lipoprotein,” “chronic periodontitis,” and “oxidative stress.” During this period, the research focus deepened, exploring specific pathological mechanisms. Keywords such as “endothelial dysfunction” and “systemic inflammation” suggest that researchers were investigating how periodontal disease influences atherosclerosis through inflammatory and immune responses.

Recent Research (2016–2023): Primary keywords include “gut microbiota,” “oral microbiome,” “chronic inflammation,” “stroke,” and “obesity.” At this stage, research hotspots further expanded into the realms of microbiota and chronic inflammation. Keywords such as “gut microbiota” and “oral microbiome” indicate that researchers are exploring the bridging role of microbiota in the relationship between periodontal disease and atherosclerosis. Additionally, keywords like “chronic inflammation” and “obesity” reflect a growing interest in systemic health factors.

From early studies on fundamental relationships to mid-term mechanistic research, and finally to recent investigations into microbiota and systemic health factors, the evolution of keywords demonstrates the deepening and broadening of research in this field. This progression reflects researchers’ increasing understanding of the complex interplay between periodontal disease and atherosclerosis.

### 3.4 Co-cited document and journal

**Figure 13:**
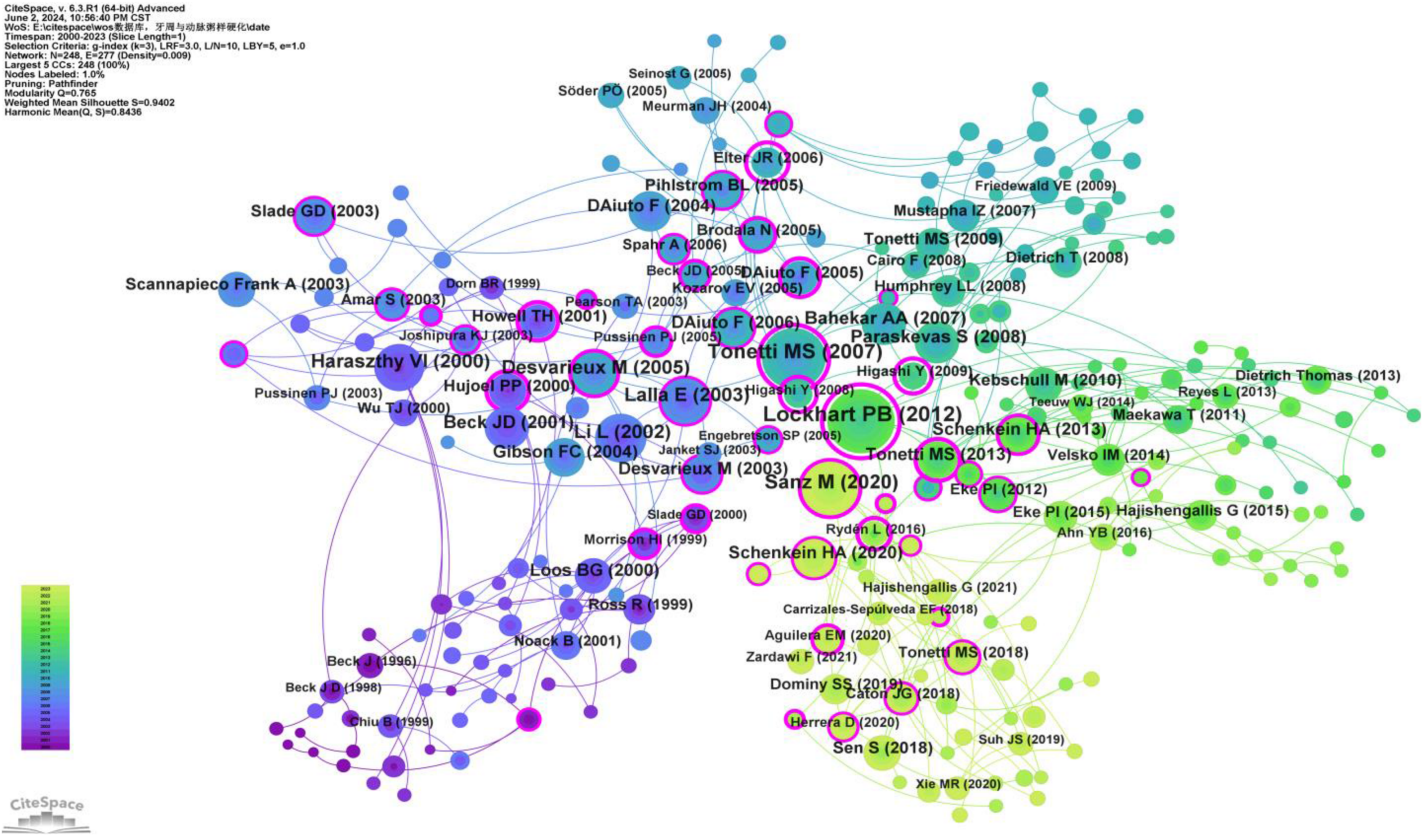
Co-cited network map of documents. After normalizing the sample literature (1,572 publications), the data were imported into CiteSpace for analysis. The selected time period spanned from 2000 to 2023, with the analysis focusing on “documents” to construct a co-citation network map of literature related to the relationship between PD and AS. Node size represents the number of citations each document received, while links between nodes indicate that the documents were co-cited in the same paper. Different colors represent the years of citation. The outermost purple ring denotes the level of centrality, with high centrality nodes considered pivotal in the research field. The network parameters are as follows: g-index (k = 3), *N* = 248 (number of network nodes), E = 277 (number of connections), and density = 0.0009 (network density).

Co-citation analysis identifies the core literature and research hotspots within a specific field by recognizing frequently co-cited documents. Utilizing CiteSpace software with a g-index set to 3, we screened out 248 co-cited documents (Figure 13). The top three most cited documents are: Lockhart PB, 2012, Circulation (98 citations) (19); Tonetti MS, 2007, The New England Journal of Medicine (90 citations) (20); and Sanz M, 2020, J Clin Periodontol (72 citations) (21). Additionally, two articles have a betweenness centrality (BC) greater than 1.00: Lockhart PB, 2012, Circulation (BC = 1.16) (19) and Elter JR, 2006, AM Heart J (BC = 1.11) (22) (Supplementary Table S11). Lockhart PB, 2012, Circulation (19) stands out as both the most cited and the article with the highest BC, indicating its broad recognition and influence among scholars in this field.

**Figure 14:**
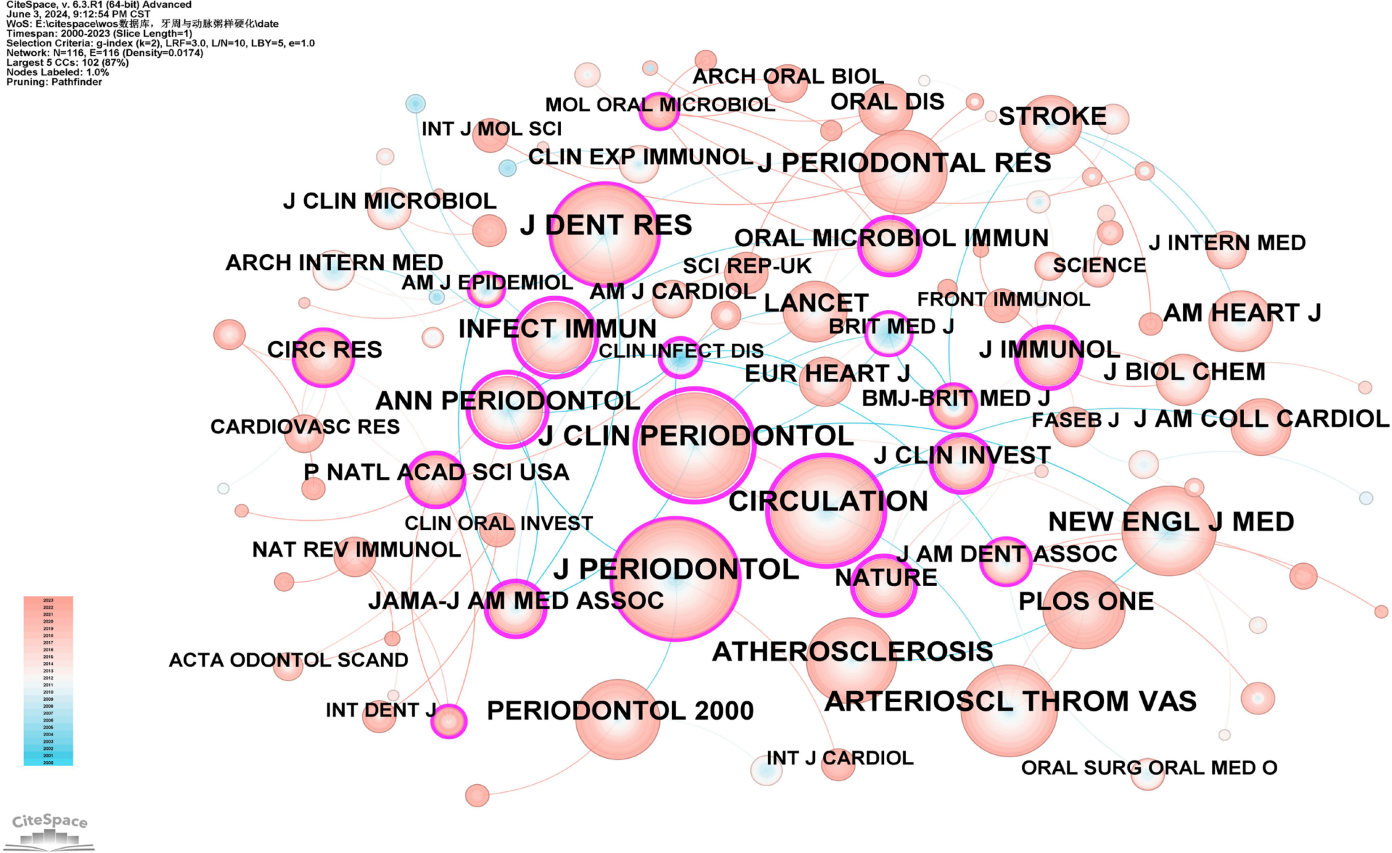
Co-cited network map of journals. After normalizing the sample literature (1,572 publications), the data were imported into CiteSpace for analysis. The selected time period spanned from 2000 to 2023, with the analysis focusing on “journals” to construct a co-citation network map of journals related to the relationship between PD and AS. Node size represents the number of citations each journal received, while links between nodes indicate co-citation in the same paper. Different colors represent the years of citation. The outermost purple ring denotes the level of centrality, with high-centrality nodes considered pivotal in the research field. The network parameters are as follows: g-index (k = 2), *N* = 116 (number of network nodes), E = 116 (number of connections), and Density = 0.0174 (network density).

Co-citation analysis of journals identifies core journals and academic dissemination channels within a specific research field by analyzing the frequency with which journals are co-cited. Using CiteSpace software with a g-index of 2, we identified 116 journals (Figure 14), The JCR categories, impact factors, and quartiles of these journals are listed in Supplementary Table S13. Based on their citation frequency and respective research areas, they were categorized into core journals in periodontal disease research and cardiovascular research. The core journals in periodontal disease research are the Journal of Periodontology, Journal of Clinical Periodontology, Journal of Dental Research, Journal of Periodontal Research, and Periodontology 2000. The core journals in cardiovascular research are Circulation and Arteriosclerosis, Thrombosis, and Vascular Biology. Their high citation frequencies indicate that these journals have significant influence in their respective fields (Supplementary Table S12).

## 4 Discussion

### 4.1 General information

This study systematically analyzed the research on the association between periodontal disease (PD) and atherosclerosis (AS) using bibliometric methods. This analysis not only identified research hotspots and trends but also provided important insights into future research directions. From 2000 to 2023, there has been a significant growth trend in research on the relationship between PD and AS, particularly after 2002, as shown in Figure 2. The increase in the number of studies reflects the academic community's growing interest in the PD-AS association. This trend may be related to the increasing recognition of the critical role of chronic inflammation and infection in the development of atherosclerosis.

Scientific collaboration is defined as researchers working together with the common goal of generating new scientific knowledge. Such collaboration occurs at various levels, including interpersonal, inter-institutional, and international cooperation. In assessing the importance of nodes in a collaboration network, both social connectivity and academic output are crucial, with betweenness centrality (BC) serving as an essential metric for evaluating social connectivity. A node with a BC exceeding 0.1 is considered a central node, relatively important, and significantly influential in the research field (23).

At the national level, as shown in Figure 3 and Supplementary Table S1, the United States (*n* = 476), Japan (*n* = 189), and China (*n* = 181) play major roles in PD and AS relationship research. The United States leads not only in the number of publications but also hosts institutions with the highest number of publications (University of North Carolina, 74) and the highest BC (Boston University, 0.47) (Figure 5, Supplementary Tables S3, S4), indicating its significant influence in this field.

Norway, however, has the highest BC (0.68) in the national collaboration analysis (Supplementary Table S2), far exceeding the United States (0.41), which has the most publications. Possible reasons for this include: Ⅰ.World-class Research Infrastructure: Norway has world-class research infrastructure and equipment, providing a solid foundation for high-quality scientific work. Norwegian universities and research institutions, such as the University of Oslo and the University of Bergen, have advanced laboratories and research facilities that enable scientists to conduct cutting-edge research. Ⅱ.High GDP: As a developed country, Norway had a per capita GDP of approximately $75,000 in 2023. Ⅲ.Comprehensive Health Data System: Norway has a comprehensive health data system that provides rich research resources for AS studies. The National Health Registries and large cohort studies like the HUNT Study offer researchers a wealth of valuable data, allowing them to conduct large-scale epidemiological research.

Most of Norway's collaborators are developed countries, with few developing countries involved. This may be due to the low priority of oral health care in developing countries, hindering the collection of PD data and research on the relationship between PD and AS (24). Additionally, compared to developing countries, developed countries provide more research funding and better infrastructure for researchers, which could also be a contributing factor (25).

In our analysis (Supplementary Tables S5–S7, Figures 5, 6), Offenbacher, Steven had the highest number of publications (*n* = 40), followed by Beck, James D (*n* = 28). Beck, James D also had the highest number of co-citations (*n* = 548). Among the top 10 co-cited authors, six played significant bridging roles (centrality >0.1). Notably, among the top 10 authors by publication count, Beck, James D and Offenbacher, Steven had extensive collaboration, while the other authors collaborated less frequently. The top 10 most cited authors had more collaboration compared to the top 10 authors by publication count.

The co-citation analysis of journals revealed that the most cited journals in this field are the Journal of Periodontology (IF = 4.3), Journal of Clinical Periodontology (IF = 6.7), and Circulation (IF = 37.8). These journals have significant influence in the field, reflecting the interdisciplinary nature of PD and AS research.

### 4.2 Knowledge base

Co-citation analysis is a method for assessing the degree of association between scientific papers. A knowledge base comprises the collection of co-cited references (11). This study includes the top 10 most co-cited papers related to the “relationship between PD and AS” (Supplementary Table S11). The most cited paper is “Periodontal Disease and Atherosclerotic Vascular Disease: Does the Evidence Support an Independent Association?” by Lockhart PB et al., published in Circulation in 2012, with 98 citations (19). The key points of this study are: 1. Shared Risk Factors: PD and ASVD (Atherosclerotic Vascular Disease) share several common risk factors, including smoking, age, and diabetes. 2. Systemic Inflammation: PD is associated with increased systemic inflammation, a recognized risk factor for ASVD. 3. Inflammatory Markers: Periodontal disease can lead to elevated levels of systemic inflammatory markers such as C-reactive protein (CRP), tumor necrosis factor-alpha (TNF-α), and various interleukins (IL-1, IL-6, IL-8). 4. Molecular Mimicry: Immune responses to periodontal pathogens may cross-react with host tissues, potentially linking PD and ASVD. 5. Periodontal Treatment: While periodontal treatment can reduce systemic inflammation and improve endothelial function in the short term, there is no evidence that it reduces the risk of ASVD.

In summary, the article concludes that although there is an association between periodontal disease and atherosclerotic vascular disease, current evidence does not support a causal relationship. Further research is needed to elucidate the potential mechanisms between these diseases and to determine whether periodontal treatment can impact cardiovascular outcomes.

The second most co-cited paper is “Treatment of Periodontitis and Endothelial Function,” by Tonetti MS et al., published in The New England Journal of Medicine in 2007 (20). This study randomly assigned 120 patients with severe periodontitis to receive either community periodontal care or intensive periodontal treatment. Endothelial function was assessed by measuring the diameter of the brachial artery during flow-mediated dilation (FMD) and evaluating inflammatory and coagulation markers before treatment and at 1, 7, 30, 60 and 180 days post-treatment. The study found:

1. Endothelial Function: 24 h after treatment, the FMD in the intensive treatment group was significantly lower than in the control group. However, at 2 and 6 months post-treatment, the FMD in the intensive treatment group was significantly higher than in the control group.
2. Inflammatory Markers: 24 h after treatment, levels of CRP, IL-6, soluble E-selectin, and von Willebrand factor were significantly elevated in the intensive treatment group. However, at 2 and 6 months post-treatment, soluble E-selectin levels in the intensive treatment group were significantly lower than in the control group.
3. Periodontal Health Improvement: The intensive treatment group showed significant reductions in plaque, gingival bleeding, and periodontal pockets at 2 and 6 months post-treatment.

Overall, this study suggests that intensive periodontal treatment can improve endothelial function and reduce levels of certain inflammatory and coagulation markers. These findings support the importance of periodontal health for cardiovascular health and suggest that clinicians should consider the potential systemic health impacts when treating periodontal disease.

The third most co-cited paper is “Periodontitis and cardiovascular diseases: Consensus report” by Sanz M et al., published in J Clin Periodontol in 2020 (21). This study aimed to update and evaluate the epidemiological association between periodontal disease and cardiovascular disease (CVD), the mechanistic links, and the impact of periodontal treatment on cardiovascular health. The main conclusions are:

1. Increased Risk: Patients with periodontal disease exhibit a significantly higher risk of cardiovascular diseases, including coronary heart disease and stroke.
2. Mechanistic Links: Evidence supports that periodontal disease influences the development of atherosclerosis through mechanisms such as systemic inflammation and bacteremia.
3. Impact of Treatment: While direct evidence for periodontal treatment reducing cardiovascular event risk is limited, treatment can significantly reduce inflammatory markers and improve surrogate indicators of cardiovascular health, such as arterial stiffness.

The fourth most co-cited paper is “Porphyromonas gingivalis Infection Accelerates the Progression of Atherosclerosis in a Heterozygous Apolipoprotein E–Deficient Murine Model” by Li L et al., published in Circulation in 2002 (26). This study used an ApoE-deficient murine model, with regular intravenous injections of live Porphyromonas gingivalis to assess its impact on the development of atherosclerotic lesions. The results are:

1. Increased Lesion Area: Mice infected with P. gingivalis showed a significant increase in the area of atherosclerotic lesions in the aorta and aortic tree compared to controls.
2. Elevated Inflammatory Mediators: P. gingivalis infection led to significantly elevated levels of systemic inflammatory mediators (IL-1β and SAA) in the serum, especially at 14 and 24 weeks.

Detection of P. gingivalis: After 24 weeks of infection, P. gingivalis 16S rRNA was detected in arterial, hepatic, and cardiac tissues.

This study demonstrates that chronic systemic infection with P. gingivalis can accelerate the development of atherosclerotic lesions in ApoE-deficient mice. It provides the first direct *in vivo* evidence that the periodontal pathogen P. gingivalis can accelerate the progression of atherosclerosis.

The fifth most co-cited paper is “Identification of Periodontal Pathogens in Atheromatous Plaques” by Haraszthy VI et al., published in J Periodontol in 2000 (27). This study analyzed 50 human specimens obtained from carotid endarterectomy to detect the presence of Chlamydia pneumoniae, human cytomegalovirus (HCMV), and bacterial 16S ribosomal RNA. The results are:

1. PCR Detection: 80% of endarterectomy specimens tested positive by PCR.
2. Viral Presence: 38% of specimens were positive for HCMV, and 18% were positive for Chlamydia pneumoniae.
3. Bacterial Presence: 72% of specimens were positive for bacterial 16S rDNA, with 44% containing at least one target periodontal pathogen.
4. Pathogen Distribution: Among periodontal pathogen-positive specimens, 30% tested positive for Bacteroides forsythus, 26% for P. gingivalis, 18% for Actinobacillus actinomycetemcomitans, and 14% for Prevotella intermedia.
5. Multiple Pathogens: The majority (59%) of periodontal pathogen-positive specimens contained two or more target pathogens.

The findings suggest that periodontal pathogens are present in atherosclerotic plaques and may be involved in the development and progression of atherosclerosis, potentially leading to coronary artery disease and other clinical outcomes. This indicates that chronic periodontal infection may play a significant role in the pathogenesis of atherosclerosis.

The sixth most co-cited paper is “Oral Infection With a Periodontal Pathogen Accelerates Early Atherosclerosis in Apolipoprotein E–Null Mice” by Lalla E et al., published in Arteriosclerosis, Thrombosis, and Vascular Biology in 2003 (28). This study utilized ApoE-null mice, which were periodically inoculated orally with P. gingivalis. Alveolar bone loss was assessed by microscopy, atherosclerotic lesions in the aortic root were quantified, serum levels of specific IgG antibodies and interleukin-6 (IL-6) were measured, and immunohistochemistry was employed to detect the expression of vascular cell adhesion molecule-1 (VCAM-1) and tissue factor. PCR was used to detect P. gingivalis DNA in aortic tissue. The results were as follows:

1. Increased Atherosclerosis: P. gingivalis infection exacerbated atherosclerosis in ApoE-null mice, with infected mice exhibiting significant alveolar bone loss indicative of periodontal infection.
2. Host Inflammatory Response: Infected mice showed widespread activation of the host inflammatory response, evidenced by elevated serum levels of IgG against P. gingivalis and increased IL-6 levels.
3. Enhanced Vascular Activation: Infected mice displayed increased expression of VCAM-1 and tissue factor in the aorta.
4. Systemic Infection Impact: Although P. gingivalis infection accelerated atherosclerosis, no effects were observed on mouse weight, blood glucose, insulin, total cholesterol, or triglyceride levels.

This study demonstrates that chronic systemic infection with P. gingivalis can accelerate the development of atherosclerotic lesions in ApoE-null mice, supporting the concept of a unified mechanism linking periodontal disease and atherosclerosis. Understanding these mechanisms is crucial for designing strategies to prevent and treat atherosclerosis and its complications.

The seventh most co-cited paper is “Periodontal Microbiota and Carotid Intima-Media Thickness” by Desvarieux M et al., published in Circulation in 2005 (29). This study analyzed 657 dentate participants out of 1,056 subjects (mean age 69 years) with no history of stroke or myocardial infarction. A total of 4,561 subgingival plaque samples were collected, and 11 known periodontal bacteria were quantitatively assessed using DNA-DNA checkerboard hybridization. Extensive cardiovascular risk factors were measured, and carotid intima-media thickness (IMT) was assessed using high-resolution B-mode ultrasound. The findings were:

1. Total Bacterial Load and IMT: The total burden of periodontal bacteria was associated with carotid IMT. Adjusted mean IMT increased with the dominance of etiologic bacteria (*P* < 0.002).
2. White Blood Cell Count: White blood cell counts increased with the burden of etiologic bacteria (*P* < 0.01), although CRP levels were not associated with periodontal microbial status (*P* < 0.82).
3. Impact of Specific Bacteria: Specific etiologic bacterial clusters had the most significant impact on IMT, with the burden and dominance of these bacteria significantly associated with increased IMT (*P* < 0.03).
4. Non-Etiologic Bacteria: Only a few non-etiologic bacteria (e.g., Micromonas micros) enhanced the relationship with IMT under specific conditions.

This study provides evidence of a direct relationship between periodontal microbiota and subclinical atherosclerosis, independent of CRP. These findings support the hypothesis that oral infections may contribute to cardiovascular disease, emphasizing the role of specific etiologic bacteria in this process.

The eighth most co-cited paper is “A Systematic Review and Meta-analyses on C-reactive Protein in Relation to Periodontitis” by Paraskevas S et al., published in J Clin Periodontol in 2008 (30). This systematic review and meta-analysis confirmed that plasma CRP levels in patients with periodontitis are higher than those in healthy controls and that periodontal treatment can partially reduce CRP levels. These findings underscore the connection between periodontal health and systemic inflammatory status, further supporting the potential impact of periodontitis on cardiovascular disease risk.

The ninth most co-cited paper is “Relationship of Periodontal Disease to Carotid Artery Intima-Media Wall Thickness” by Beck JD et al., published in Arteriosclerosis, Thrombosis, and Vascular Biology in 2001 (31). This cross-sectional study analyzed the relationship between carotid intima-media thickness (IMT) and the severity of periodontitis. The sample included 573 participants, with periodontitis severity assessed through periodontal examination and IMT measured using carotid ultrasound. The results indicated:

1. Severe Periodontitis and IMT: Severe periodontitis was significantly associated with increased carotid IMT. Participants with severe periodontitis had higher IMT than those with no or mild periodontitis.
2. Increased Risk of Thickened IMT: Severe periodontitis increased the odds of having a thickened IMT (≥1 mm) by 1.3 times (adjusted OR 1.31, 95% CI 1.03–1.66).
3. Independent Risk Factor: Severe periodontitis remained a significant risk factor for increased IMT even after adjusting for other traditional atherosclerosis risk factors (e.g., age, sex, LDL cholesterol, diabetes, hypertension, smoking).
4. Dose-Response Relationship: There was a dose-response relationship between the severity of periodontitis and increased IMT; as the severity of periodontitis increased, IMT also increased.
5. Public Health Significance: Periodontitis may play a critical role in the development of atherosclerosis.

Given that periodontitis is preventable and treatable, this finding has important public health implications.

The tenth most co-cited paper is “The Prevalence and Incidence of Coronary Heart Disease is Significantly Increased in Periodontitis: A Meta-analysis” by Bahekar AA et al., published in the American Heart Journal in 2007 (32). This meta-analysis systematically reviewed studies from PubMed, Cochrane, EMBASE, and CINAHL databases that met inclusion criteria (sample size ≥80, well-defined periodontitis and coronary heart disease, sufficient statistical data). Exclusion criteria included duplicate studies and animal experiments. Data collected included sample size, study design, population characteristics, follow-up duration, and outcomes. The Mantel-Haenszel fixed-effect model was used to calculate relative risk (RR) and odds ratio (OR), with heterogeneity tests conducted. The results showed:

1. Prospective Cohort Studies: Five studies (86,092 patients) indicated that periodontitis increased the risk of coronary heart disease by 1.14 times (95% CI 1.074–1.213, *P* < .001).
2. Case-Control Studies: Five studies (1,423 patients) showed that periodontitis increased the risk of coronary heart disease by 2.22 times (95% CI 1.59–3.117, *P* < .001).
3. Cross-Sectional Studies: Five studies (17,724 patients) showed that periodontitis increased the risk of coronary heart disease by 1.59 times (95% CI 1.329–1.907, *P* < .001).
4. Tooth Loss and Risk: Patients with fewer than 10 teeth had a 1.24 times higher risk of coronary heart disease (95% CI 1.14–1.36, *P* < .0001).

This meta-analysis indicates a significant increase in the prevalence and incidence of coronary heart disease in patients with periodontitis. Periodontitis may be a risk factor for coronary heart disease, necessitating further prospective studies to validate this association and evaluate the impact of periodontal treatment on reducing coronary heart disease risk.

In summary, the top 10 most co-cited papers consistently suggest a significant association between periodontal disease (PD) and atherosclerosis (AS), with evidence that periodontal treatment can potentially reduce the risk of atherosclerosis. These findings highlight the importance of periodontal health management as a potential strategy for mitigating systemic inflammation and reducing cardiovascular disease risk. Further research is essential to elucidate the underlying mechanisms and to develop effective preventive and therapeutic interventions.

### 4.3 Research hotspots between PD and AS

Keywords serve as indicators of research hotspots and directions within a specific field. According to Supplementary Table S8, the top 25 keywords each appear more than 70 times, highlighting the research hotspots in the relationship between periodontal disease (PD) and atherosclerosis (AS). Representative keywords include Porphyromonas gingivalis, inflammation, risk, oral health, C-reactive protein, atherosclerosis risk, and endothelial dysfunction. These keywords collectively provide a comprehensive overview of the research landscape in this domain.

Periodontal disease (PD) and atherosclerosis (AS) share multiple common risk factors, including smoking, diabetes, hypertension, hyperlipidemia, genetic predisposition, and age. Among these, smoking is likely the most significant risk factor (33). Smoking has been shown to affect various aspects of the host immune response (34). From a biological perspective, smoking can adversely impact fibroblast function (35), neutrophil chemotaxis and phagocytosis (36), immunoglobulin production (37, 38), and induce peripheral vasoconstriction (39). Consequently, smoking exhibits potential pathogenic properties that may contribute to the development of both atherosclerosis and periodontal disease.

Periodontal disease (PD) is an inflammatory condition primarily caused by Porphyromonas gingivalis (P.g), a Gram-negative anaerobic bacterium commonly found in the human oral cavity, particularly in subgingival tissues and periodontal pockets (40). P.g plays a significant role in the association between periodontal disease and atherosclerosis. It can enter the bloodstream and disseminate to atherosclerotic plaques, directly infecting and exacerbating their formation (6, 41, 42). Additionally, P.g produces various toxins, such as Lipopolysaccharides (LPS), which can travel through the bloodstream to the vascular endothelium, causing direct damage and dysfunction of endothelial cells (43, 44). Furthermore, P.g can activate the host immune system, triggering immune responses that further damage endothelial cells and atherosclerotic plaques (26). Endothelial dysfunction is an early and critical step in the development of atherosclerosis (45).

Periodontal disease (PD) is associated with increased systemic inflammation, which is a recognized risk factor for atherosclerosis (AS). PD can elevate levels of systemic inflammatory markers such as C-reactive protein (CRP) and various interleukins (e.g., IL-1, IL-6, IL-8) (19). These inflammatory markers can damage vascular endothelial cells, inducing or exacerbating endothelial dysfunction. CRP is identified as an acute-phase plasma protein primarily expressed and secreted by the liver (46, 47). Its concentration increases during tissue injury, infection, and chronic inflammation (46). Growing evidence indicates that CRP plays a significant role in inflammation and the host response to infection, including the complement pathway, apoptosis, phagocytosis, nitric oxide release, and the release of inflammatory cytokines, particularly interleukin-6 and tumor necrosis factor-alpha (48). Additionally, numerous studies have reported a correlation between elevated CRP levels and increased AS risk (4, 49). Paraskevas S et al. suggest that periodontal treatment can partially reduce CRP levels, underscoring the importance of periodontal therapy (30).

According to Figure 12, research on the association between periodontal disease (PD) and atherosclerosis (AS) has evolved from early foundational studies to mechanistic investigations and more recently to studies on microbiota and systemic health factors. The evolution of keywords reflects the deepening and expansion of this research field, demonstrating a gradual understanding of the complex relationship between PD and AS. Future research should focus on elucidating the underlying mechanisms between PD and AS, such as the intricate relationship between PD and endothelial dysfunction. Multidisciplinary collaboration and the application of innovative technologies will provide new insights and methods for preventing and treating both diseases. This approach will not only improve oral and cardiovascular health but also reduce the incidence and mortality of these related diseases, thereby enhancing overall public health.

### 4.4 Strengths and limitation

Consistent with other bibliometric analyses, this study has several limitations: (1) CiteSpace cannot fully replace systematic searches; (2) This study only included English-language research published between 2000 and 2023, which may have led to the exclusion of some key studies; (3) All data were retrieved and downloaded from the WoSCC database, potentially missing some papers not included in this database. However, WoSCC is the most commonly used database in bibliometrics analyses, and its data can represent a substantial portion of the available information; (4) Some recently published high-quality studies may have been excluded due to insufficient citation counts, potentially affecting the identification of research hotspots and emerging trends in this study. Despite these limitations, we believe this study effectively captures the research hotspots and emerging trends in PD and AS research. Based on this study, future researchers can quickly understand the current state of the field and select research directions according to the identified hotspots and emerging trends.

## 5 Conclusion

An increasing body of basic research and clinical practice supports the association between periodontal disease (PD) and atherosclerosis (AS). Bibliometric analysis provides an objective, quantitative method for evaluating the development trends and frontiers in the field of PD and AS. Over the past twenty-four years, the number of publications and citations in this field has been on the rise, indicating growing interest and research enthusiasm among scholars. Globally, the United States leads in this research area. Moving forward, enhancing collaboration and communication between institutions and authors is crucial. Key research hotspots in this field include Porphyromonas gingivalis, inflammation, risk, oral health, C-reactive protein, atherosclerosis risk, and endothelial dysfunction. Additionally, elucidating the underlying mechanisms between PD and AS is emerging as a new research trend. This study summarizes the knowledge base in this field, reveals current research hotspots and emerging trends, and provides valuable guidance for future researchers in selecting appropriate research directions.

## Data Availability

The original contributions presented in the study are included in the article/Supplementary Material, further inquiries can be directed to the corresponding author.
